# Assessment of depressive symptoms in hospitalized patients with schizophrenia

**DOI:** 10.1192/j.eurpsy.2022.2030

**Published:** 2022-09-01

**Authors:** M. Abdelkefi, S. Ellouze, S. Ajmi, M. Turki, N. Halouani, J. Aloulou

**Affiliations:** 1 CHU Hedi Chaker, Psychiatry, sfax, Tunisia; 2 Hedi Chaker University Hospital, Psychiatry B, Sfax, Tunisia; 3 Hedi Chaker University Hospital, Psychiatry “b” Department, Sfax, Tunisia; 4 Hedi Chaker University Hospital, Psychiatry, Sfax, Tunisia

**Keywords:** schizophrénia, Depression, decompensation, acute

## Abstract

**Introduction:**

The prevalence of depressive disorders in patients with schizophrenia is estimated at 25%. Nevertheless, depressive symptoms occurring during the acute decompensation of schizophrenia have rarely been studied.

**Objectives:**

The aim of our study was to assess depressive symptoms in hospitalized patients suffering from schizophrenia.

**Methods:**

We conducted a cross-sectional, descriptive and analytical study, including 30 schizophrenic patients, hospitalized in the psychiatry B “department of the Hedi Chaker university hospital in Sfax. The assessment of clinical severity of schizophrenia was performed by the Positive and Negative Syndrome Scale (PANSS), that of depressive symptoms by the “Calgary Depression Scale for Schizophrenia” (CDSS).

**Results:**

The mean age of patients was 41.2. Most of patients were male (86.7%) and unemployed (83.3%). Only 13.3% of them were married. Patients were hospitalized 8.83 times in average. A personal history of suicide attempts was found in 16.70% of cases. The mean score in the CDSS scale was 5.12. According to the CDSS score, a depressive state was diagnosed in 36.7% % of patients. Depression was associated with significantly more frequent history of suicide attempts (p=0.028), as well as significantly higher scores in the positive dimension of the PANSS (p=0.03).

**Conclusions:**

Our results show that depressive symptoms are common during the acute decompensation phase of schizophrenia. They are associated with impaired functioning of patients, as well as a higher risk of suicide. Screening for depressive symptoms in patients hospitalized for schizophrenia is therefore essential in order to ensure better management.

**Disclosure:**

No significant relationships.

